# Baseline Cognitive Performance Moderates the Effects of Physical Activity on Executive Functions in Children

**DOI:** 10.3390/jcm9072071

**Published:** 2020-07-01

**Authors:** Toru Ishihara, Eric S. Drollette, Sebastian Ludyga, Charles H. Hillman, Keita Kamijo

**Affiliations:** 1 Graduate School of Human Development and Environment, Kobe University, Kobe 657-8501, Japan; tishihara@people.kobe-u.ac.jp; 2 Department of Kinesiology, University of North Carolina at Greensboro, Greensboro, NC 27413, USA; esdrolle@uncg.edu; 3 Department of Sport, Exercise and Health, University of Basel, 4052 Basel, Switzerland; sebastian.ludyga@unibas.ch; 4 Department of Psychology, Department of Physical Therapy, Movement, & Rehabilitation Sciences, Northeastern University, Boston, MA 02115, USA; c.hillman@northeastern.edu; 5 University of Tsukuba, Tsukuba 305-8577, Japan

**Keywords:** exercise, fitness, executive functions, cognitive control, adolescent

## Abstract

Findings regarding the effects of regular physical activity on cognition in children have been inconsistent due to a number of demographic factors and experimental considerations. The present study was designed to examine baseline cognitive performance and executive function demands, as possible factors underlying the lack of consensus in the literature, by investigating the moderating role of those factors on the effects of physical activity on cognition. We reanalyzed data from three randomized controlled trials, in which the effects of regular physical activity intervention on cognition were examined using executive function tasks that included at least two task conditions requiring variable executive function demands, with a cumulative total of 292 participants (9–13 years). The results indicate that cognitive improvements resulting from physical activity intervention were greater in children with lower baseline cognitive performance. The main analysis revealed that beneficial effects of physical activity intervention on cognitive performance were generally observed across executive function conditions. However, secondary analyses indicated that these general effects were moderated by baseline performance, with disproportionately greater effects for task conditions with higher executive function demands. These findings suggest that baseline cognitive performance is an individual difference variable that moderates the beneficial effects of physical activity on executive functions.

## 1. Introduction

There is growing evidence that regular physical activity (PA) is an efficacious and low-cost health behavior that supports cognitive and brain development in children and adolescents [1,2]. Indeed, previous randomized controlled trials (RCTs) have shown that PA intervention increases brain-derived neurotrophic factor, vascular endothelial growth factor and insulin-like growth factor-1 levels in the serum [3], as well as improves brain structural and functional integrity [4,5], and that these changes might, in turn, result in improvements in executive functions (EFs) [5,6,7,8,9,10] in preadolescent and adolescent children. EFs refer to a subset of cognitive operations that underlie goal-directed actions including inhibition (the ability to suppress impulses and natural, habitual or dominant behavioral responses), working memory (the ability to hold and process new and already stored information) and cognitive flexibility (the ability to switch perspectives or one’s focus of attention) [11,12]. It has been well documented that EFs are closely associated with academic performance [13,14], indicating any relationship between PA and EFs might be especially important to the effective functioning of school aged children.

However, the effects of regular PA on EFs and academic performance in children have been inconsistent in the literature. A recent systematic review showed that approximately one-third of studies reported no regular PA effects [15]. For example, a large cluster RCT [16] examined the effects of a seven-month school-based PA intervention on academic performance in preadolescent children and found no overall effect of the PA intervention. However, subgroup analyses revealed that children in the lowest tertile group for math performance at baseline exhibited the largest improvement in academic achievement following the intervention. No such beneficial effect of the PA intervention was observed for the higher tertile groups. Similarly, Bartee, Heelan and Dority [17] indicated that the beneficial improvements in aerobic fitness on math performance were stronger for lower performing math students. Across studies, these findings suggest that children who perform more poorly at baseline are more likely to exhibit larger PA-induced improvements in academic performance following PA intervention, whereas a similar beneficial response to PA intervention appears limited in children who exhibit higher baseline performance. Several studies further showed that the beneficial effects of acute PA intervention on EF performance were greater for lower baseline performers [18,19]. Collectively, based on this body of evidence, it is reasonable to suggest that the effects of regular PA on EFs would vary as a function of baseline performance. The present study examined baseline performance as one possible factor for the lack of consensus in the literature by investigating the moderating role of baseline performance on the effects of regular PA intervention on EF performance. Understanding the role of baseline performance on the effect of PA on EFs is important to gain insights on whether PA has a normalizing or enhancing effect on this cognitive domain. Such insights are necessary to identify children who can benefit from PA and those who require other types of intervention.

Poorer baseline cognitive performance in children might reflect lower cognitive ability, which is caused by factors such as delayed cognitive maturation and low socioeconomic status [20]. From a different perspective, baseline performance should also reflect the relative task difficulty for the individual, given that cognitive ability varies across individuals. That is, task difficulty is an important variable that may influence not only cognitive performance, but also the susceptibility of cognitive performance to change following intervention. For example, a longitudinal study [21] examined the effects of a twelve-month sport participation program (low frequency vs. high frequency) on EFs and found that the beneficial effects of sport participation were observed for working memory performance but not for inhibitory control and cognitive flexibility performance, an effect that differs from other published PA trials [7]. Participants in the Ishihara and Mizuno study [21] exhibited lower baseline accuracy on a two-back working memory task (mean accuracy = 87%) compared to inhibitory control (Stroop task, mean accuracy = 97%) and cognitive flexibility (switch task, mean accuracy = 93%) tasks. Thus, if EF tasks are not sufficiently difficult, the beneficial effects of regular PA on EF performance may not be detected. Indeed, Ishihara and Mizuno [21] showed that the average baseline z-score for each EF task was strongly correlated with the effect size of sport participation (Spearman’s correlation coefficient = −0.90), indicating a ceiling effect for the easier EF tasks. Collectively, such findings suggest that the effects of regular PA intervention on EFs are altered by difficulty level, which is reflected in baseline performance, and may be considered an individual difference measure of cognitive ability.

Another related factor that affects the relationship between regular PA intervention and changes in cognition is EF demands. It has been well documented that the beneficial effects of regular PA intervention, while generalized across aspects of cognition, are selectively and disproportionately greater for tasks or task components requiring greater amounts of EFs, when compared to lower-order cognitive functions [22]. Neuroimaging studies have demonstrated that regular PA intervention changes brain activity in regions critically involved in EFs, such as the prefrontal cortex and the anterior cingulate cortex [5,6], supporting the disproportionate effects of PA intervention on EFs. Additionally, given that task conditions requiring higher EF demands (hereafter referred to as “higher-EF condition”) should result in lower baseline performance relative to task conditions necessitating lower EF demands (hereafter referred to as “lower-EF condition”), disproportionately greater effects of PA intervention would be expected for higher-EF conditions.

The present study investigated whether the effects of regular PA intervention on cognition are moderated by baseline performance and EF demand. We reanalyzed data from three RCTs [7,8,9] in which the effects of PA intervention on cognition were examined using EF tasks that included at least two task conditions requiring variable EF demands (i.e., lower-EF condition and higher-EF condition). We hypothesized that the beneficial effects of PA intervention on cognitive performance would be disproportionately greater for lower compared to higher baseline performers, as well as for all participants in the higher-EF, compared to the lower-EF, condition.

## 2. Materials and Methods

### 2.1. Participants

This study incorporated data from three RCTs that examined the effects of a PA intervention on EFs in children with a total of four cognitive tasks [7,8,9]. Table 1 shows a summary of these RCTs. The Fitness Improves Thinking in Kids (FITKids) trial [7,8] and the Active Breaks and Executive Function (ACTIBrEX) trial [9] were registered at www.clinicaltrials.gov (NCT01334359) and the German Clinical Trials Register (DRKS00013225), respectively. Given the sample size needed to achieve the necessary power to detect moderators can be very high [23], this reanalysis combined datasets with the aim of overcoming the issue of limited power. All procedures performed in studies involving human participants were in accordance with the ethical standards of the local ethics committees of the University of Illinois at Urbana-Champaign and the Ethikkommission Nordwest- und Zentralschweiz and with the 1964 Helsinki declaration and its later amendments or comparable ethical standards. Informed assent and consent were obtained from all individual participants and their legal guardians included in each study. Additional informed consent was obtained from all individual participants for whom identifying information is included in this article.

### 2.2. Cognitive Tasks

Here, we only provide brief descriptions of the cognitive tasks, which are essential for understanding the present analysis. However, greater detail of each task is provided in the original references [7,8,9]. All three studies assessed both response accuracy and reaction time for each task. The effects of the PA intervention were observed for response accuracy by Hillman et al. [7] and Kamijo et al. [8], whereas the effects were found for reaction time by Ludyga et al. [9]. This difference is likely a result of differences in the nature of the EF tasks employed and/or between study differences in participant age. The present study was designed not to examine whether PA intervention improves EFs, but rather to test whether the beneficial effects of PA intervention on cognitive performance are moderated by baseline performance and EF demands. Based on this purpose, we examined cognitive performance broadly, including either accuracy or reaction time as a function of the outcome influenced by the PA intervention in each study. These main outcomes were converted to z-scores after controlling for age and sex. Positive scores in each task indicate better performance (i.e., higher accuracy or shorter reaction time).

#### 2.2.1. Modified Flanker Task

In the study of Hillman et al. [7], a modified flanker task [24] had participants press either a left or right button corresponding to the direction of a centrally presented target fish amid an array of flanking fish, which faced either in the same direction (congruent condition) or the opposite direction (incongruent condition) of the target fish. The incongruent condition engenders higher EF demands because of the need to inhibit incorrect response activation generated by the flanking stimuli. For the present analysis, we defined the congruent and incongruent conditions as the lower-EF and higher-EF conditions, respectively.

#### 2.2.2. Color-Shape Switch Task

In the study of Hillman et al. [7], a color-shape switch task [25] had participants press either the left or right button corresponding to the shape (square or circle) or color (blue or green) of presented characters. This task consisted of a homogeneous task condition, in which a single task (i.e., shape or color) was repeated, and a heterogeneous condition, in which the two tasks were randomly interleaved with equal probability. The heterogeneous task condition involves higher EF demands due to the need to maintain multiple task sets active in working memory and the requisite inhibition of a task set on switch trials [26,27]. For the present analysis, we defined the homogeneous and heterogeneous task conditions as the lower-EF and higher-EF conditions, respectively.

#### 2.2.3. Modified Sternberg Task

In the study of Kamijo et al. [8], a modified Sternberg task [28] asked participants to encode a memory set containing an array of one, three, or five letters and press either the left or right button corresponding to whether a subsequently presented single probe letter was present or absent from the encoded letter array. Increased EF demands are required for larger set sizes. For the present analysis, we defined the one-letter and five-letter conditions as the lower-EF and higher-EF conditions, respectively.

#### 2.2.4. Modified Stroop Task

In the study of Ludyga et al. [9], a modified Stroop task [29] asked participants to press one of three buttons corresponding to the ink color of a color word (“blue”, “green” and “yellow”). A color word was presented in the same color (congruent condition, e.g., “yellow” displayed in yellow) or in a different color (incongruent condition, e.g., “green” displayed in yellow). The incongruent condition requires higher EF demands to inhibit the prepotent response to read the word and instead name the color. For the present analysis, we defined the congruent and incongruent conditions as the lower-EF and higher-EF conditions, respectively.

### 2.3. Statistical Analysis

To test the moderation effects of baseline performance and EF demands on the relationship between PA intervention and changes in cognitive performance, we used hierarchical multiple regression analysis. The pre–post changes in cognitive performance were taken as the dependent variable. The group (coded as control = 0, intervention = 1), baseline performance, baseline performance^2^ (square value of baseline performance) and EF condition (coded as lower = 0, higher = 1) were entered as independent variables in Step 1, and two- to three-way interactions were entered as independent variables in Steps 2 and 3, respectively. Baseline performance was investigated by assessing linear (i.e., baseline performance) and curvilinear (i.e., baseline performance^2^) relationships with pre–post change scores. Based on our a priori hypothesis, post hoc analyses were performed only when interactions involving group were significant. All statistical analyses were conducted with α = 0.05 using R Studio software version 1.1.463 (R Studio, Inc., Boston, MA, US).

## 3. Results

### 3.1. Main Analysis

**Figure 1:**
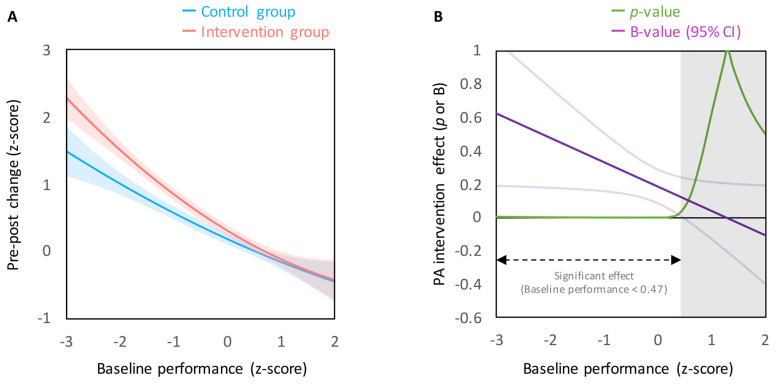
(**A**) Relationship between baseline performance and pre–post changes in cognitive performance, illustrating the group × baseline performance interaction. Regression lines are shown with 95% confidence bands (shaded areas). (**B**) Relationship between baseline performance and the PA (physical activity) intervention effects. The PA intervention effects were statistically significant if baseline performance was lower than mean +0.47 *SD*.

The results of hierarchical multiple regression predicting the pre–post change in cognitive performance are summarized in Table 2. This hierarchical multiple regression demonstrated significant main effects of group (β = 0.09, 95% confidence interval [CI] = 0.04 to 0.15, p < 0.001), baseline performance (β = −0.57, 95% CI = −0.64 to −0.50, p < 0.001), baseline performance^2^ (β = 0.06, 95% CI = 0.002 to 0.13, p = 0.04),and EF condition (β = −0.17, 95% CI = −0.22 to −0.11, p < 0.001) in Step 1 with a significant R^2^ (R^2^ = 0.33, p < 0.001). Step 2 revealed significant two-way interactions between group and baseline performance (β = −0.07, 95% CI = −0.14 to −0.003, p = 0.04; Figure 1A) and between baseline performance and EF condition (β = 0.07, 95% CI = 0.008 to 0.14, p = 0.03) with a significant change in R^2^ (ΔR^2^ = 0.01, p = 0.003). These results indicate that the beneficial effects of PA intervention on cognitive performance were greater in lower baseline performers irrespective of EF conditions (Figure 1A). The PA intervention effects were statistically significant when baseline performance was lower than the mean + 0.47 SD (Figure 1B).

Because the present analysis included both response accuracy and reaction time, we conducted additional analyses to examine whether the moderating roles of baseline performance and EF condition on the effects of PA intervention differ based on the specific measure (coded as response accuracy = 0, reaction time = 1). The results reveal no significant interactions of group × baseline performance × measure (β = 0.007, 95% CI = −0.05 to 0.06, *p* = 0.80) and group × EF condition × measure (β = 0.02, 95% CI = −0.03 to 0.07, *p* = 0.46). These results suggest that the moderating roles of baseline performance and EF condition did not differ between measures.

### 3.2. EF Difference Score Analysis

**Figure 2:**
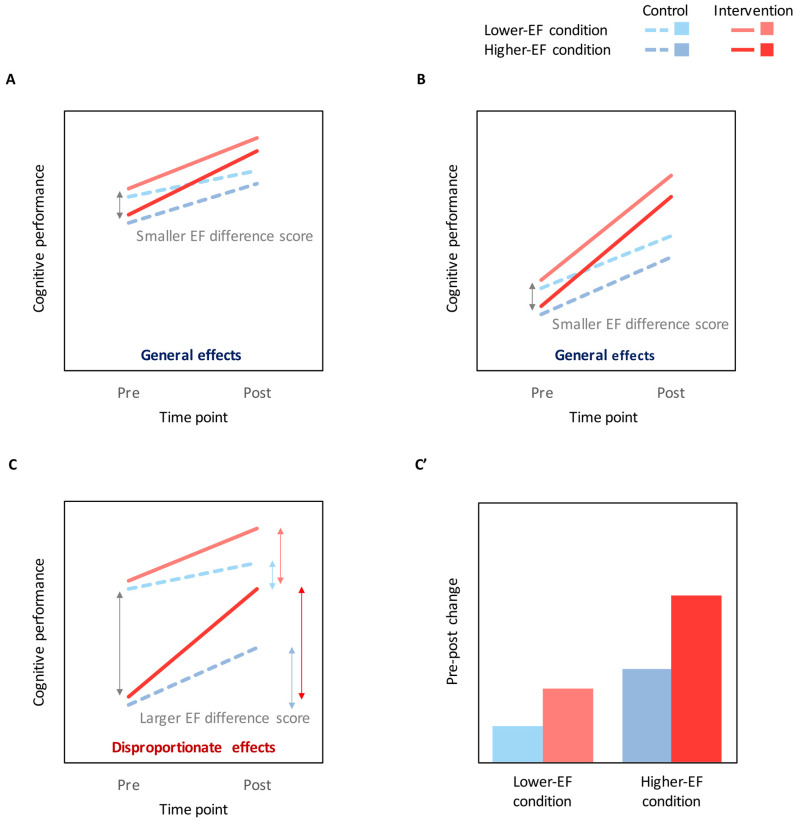
Conceptual diagrams of the prediction of changes in cognitive performance based on the baseline EF (executive function) difference scores. (**A**) If baseline cognitive performance is relatively high in both the lower-EF and higher-EF conditions (i.e., smaller EF difference score), cognitive improvements resulting from a PA intervention would be relatively small in the both EF conditions, resulting in the general effects of PA on cognitive performance irrespective of EF conditions. (**B**) If baseline cognitive performance is relatively low in the both EF conditions (i.e., smaller EF difference score), cognitive improvements resulting from a PA intervention would be relatively large in the both EF conditions, resulting in the general effects. (**C**) If the baseline EF difference score is sufficiently large, cognitive improvements resulting from a PA intervention would be greater for the higher-EF condition relative to the lower-EF condition, resulting in the disproportionate effects. Colored double-headed arrows show pre–post changes in cognitive performance (i.e., the present dependent variable) in each group and EF condition. (**C’**) The line graph in (**C**) is converted to a bar graph using the pre–post changes in cognitive performance in accord with Figure 3B.

**Figure 3:**
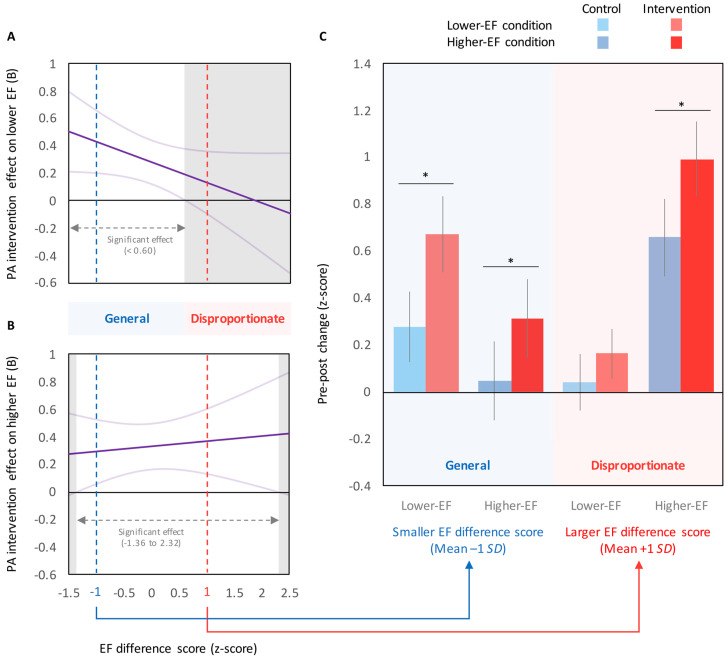
(**A**,**B**) The Johnson–Neyman regions of significance for the relationship between EF difference score and PA intervention effects (B-value ± 95% CI; A: lower EF condition; B: higher EF condition). (**C**) Typical examples of the general (mean −1 *SD*) and disproportionate (mean +1 *SD*) effects of PA intervention on EF performance. Values are presented as mean ± 95% confidence interval. * *p* < 0.05.

Contrary to our hypothesis, the beneficial effects of PA intervention on cognitive performance did not differ across EF conditions (i.e., the group × EF condition was not significant after controlling for baseline performance). Given that the main analysis indicated that lower baseline performance was associated with greater benefits from PA interventions, the disproportionate effects of PA intervention on EF performance would be predicted if the difference in cognitive performance between lower-EF and higher-EF conditions (hereafter referred to as “EF difference score”) were sufficiently large. Conceptual diagrams of this prediction are illustrated in Figure 2. Based on this prediction, we used the Johnson–Neyman technique [30] to examine the relationship between EF difference scores and PA intervention effects. In the lower EF condition (Figure 3A), PA intervention effects were significant when the EF difference score was lower than mean +0.60 *SD*. In the higher EF condition (Figure 3B), PA intervention effects were significant when the EF difference score was between mean −1.36 *SD* and mean +2.32 *SD*. These results indicate that generalized effects of PA on cognitive performance, irrespective of EF conditions, were detected when the EF difference score was lower than mean +0.60 *SD*, whereas disproportionately larger effects on a higher EF condition were detected when the EF difference score was higher than mean +0.60 *SD*. Typical examples of the general (mean −1 *SD*) and disproportionate (mean +1 *SD*) effects are illustrated in Figure 3C. These results support the above prediction that the general versus disproportionate effects of PA on cognitive performance may be altered by baseline EF difference scores.

## 4. Discussion

The main findings demonstrate that cognitive improvements resulting from regular PA intervention were greater in individuals with lower baseline cognitive performance (Figure 1A). This result is consistent with a large-scale cluster RCT, which found that the beneficial effects of a PA intervention on academic performance were only observed for lower performing students [16]. These findings leave open the possibility that such improvements may simply reflect a “catch-up” process (e.g., regression toward the mean) in lower baseline performers in the PA intervention group. The fact that, in two of the included studies [7,8], baseline performance was incidentally, but not significantly, higher for the control group relative to the intervention group may also be suggestive of these “catch-up” improvements. The observed main effect of baseline performance can be interpreted as “catching up” across both the control and intervention groups. However, this main effect was superseded by an interaction of group and baseline performance, indicating that cognitive improvements were greater for the intervention group relative to the control group (Figure 1A). Accordingly, such improvements for the intervention group must also reflect the beneficial effects of PA intervention on cognitive performance. Thus, the differential findings in the intervention group cannot be attributed to differences in development, rather such findings are attributable to the PA intervention.

Notably, the present analysis also revealed that the pre–post changes in cognitive performance did not differ between groups, even for high baseline performers (Figure 1A,B). This finding is in agreement with a recent systematic review concluding that increases in time spent in PA do not negatively affect cognitive function and academic achievement [1]. The present results further suggest the possibility that the beneficial effects of regular PA intervention would be observed for higher baseline performers, if the EF tasks employed were sufficiently difficult. In this context, the current results also account for task-dependent effects of PA intervention on EF performance that have been observed in previous studies using multiple EF tasks [10,21]. In these studies, the beneficial effects of regular PA intervention on EF performance were only observed for a relatively difficult EF task that had resulted in lower baseline performance, while such effects did not extend to “easier” EF tasks. Thus, if baseline performance is too high (i.e., the EF task is too easy or the participant exhibits high EF capacity), the beneficial effects of regular PA intervention might not be detected.

Contrary to our hypothesis, the main findings suggest a general effect of regular PA intervention on cognitive performance, irrespective of EF conditions. However, the findings from the EF difference score analysis revealed that the general versus disproportionate effects were altered depending on baseline EF difference scores (Figure 3). Specifically, generalized effects were observed when the EF difference score was relatively small, whereas disproportionately larger effects were found when the EF difference score was sufficiently large. Accordingly, we suggest that disproportionately greater effects of PA on a higher-EF condition would be confirmed only when baseline performance reaches an optimal level. Several prior child studies have shown disproportionate effects of regular PA intervention on EFs [5,7,8], whereas other studies have found more generalized effects, irrespective of EF demands [7,9,31,32]. Based on the current findings, baseline EF difference scores may be the cause of this discrepancy in the literature. Note that neuroimaging studies have shown that brain activity, such as in the prefrontal cortex and associated networks, during EF tasks change following regular PA intervention [5,6]. Accordingly, it is reasonable to suggest that the disproportionately larger effects of a PA intervention on EFs observed in previous RCTs [5,7,8] are not only due to an optimal level of baseline performance but also due to changes in brain networks associated with EFs.

There are several limitations worth noting. First, the age range is limited. During childhood, significant developmental changes occur in cortical and subcortical brain structures, while the prefrontal cortex, which plays a key role in the effective regulation of EFs, exhibits delayed maturation relative to other brain regions [33,34]. Accordingly, baseline EF performance should change dramatically based on developmental stages. Such developmental transition would likely affect the relationship between baseline performance and pre–post changes in cognition. Related to this issue, given that participants’ age differed among our RCTs, the present results should be interpreted with caution. Second, given that our RCTs [7,8,9] used different EF tasks, task difficulty within EF tasks and individual differences in baseline performance should differ among EF tasks as evidenced by various ranges of coefficient of variation (*SD*/mean: 0.08–0.30). Accordingly, the present study does not allow for us to discuss whether the moderating effects of baseline performance differ depending on the nature of the EF tasks employed. Collectively, future large-scale meta-analyses should cover a wider age range and a great number of EF tasks to address these issues. Third, the present findings cannot distinguish whether the beneficial effects of PA intervention would be selectively observed for children with lower cognitive ability independent of task difficulty or would be observable in higher baseline performers under conditions when task difficulty is challenging enough. Further studies manipulating task difficulty are required to address this potential confound. Finally, there may be several individual differences that account for lower baseline performance including demographic characteristics (e.g., low socioeconomic status), physical health (e.g., obesity) and mental health (e.g., ADHD) variables. However, the present findings do not allow for the identification of whether all of these variables are related to the effects of regular PA on EFs, or which of those variables are more likely to be susceptible to the beneficial effects.

## 5. Conclusions

The results of this study suggest that the beneficial effects of regular PA intervention on cognitive performance are greater in lower baseline performers. Further, the general versus disproportionate effects of PA intervention are likely to be altered by baseline EF difference scores, indicating that the disproportionate effects would be confirmed only when baseline performance reaches an optimal level. Collectively, the present study suggests that baseline EF performance is an individual difference variable that moderates the effects of regular PA intervention on changes in cognitive performance. Such a finding has implications for the synergistic relationship of PA and EFs during childhood and supports for the provision of regular PA opportunities during the school day.

## Figures and Tables

**Table 1:** Summary of the randomized controlled trials included in this study.

Trial	*N*	Age	Intervention	Task	Main Findings
Hillman et al., 2014	Control: 112 Intervention: 109	Control: 9 years old Intervention: 9 years old	Control: Waitlist (no intervention) Intervention: 9 months, 5 days/week, 120 min, 70+ min of moderate-to-vigorous PA	Flanker task Lower-EF: congruent Higher-EF: incongruent	PA intervention improved cognitive performance across the congruent and incongruent conditions (i.e., general effects
				Color-shape switch task Lower-EF: homogeneous Higher-EF: heterogeneous	PA intervention improved cognitive performance in the heterogeneous condition to a greater extent than in the homogeneous condition (i.e., disproportionate effects).
Kamijo et al., 2011	Control: 16 Intervention: 20	Control: 9 years old Intervention: 9 years old	Control: Waitlist (no intervention) Intervention: 9 months, 5 days/week, 120 min, 70+ min of moderate-to-vigorous PA	Sternberg task Lower-EF: one letter Higher-EF: five letters	PA intervention improved cognitive performance in the five letters condition to a greater extent than in the one letter condition (i.e., disproportionate effects).
Ludyga et al., 2018	Control: 16 Intervention: 19	Control: 12 years old Intervention: 13 years old	Control: 8 weeks, 5 days/week, 20 min of social interaction Intervention: 8 weeks, 5 days/week, 20 min of moderate PA	Stroop task Lower-EF: congruent Higher-EF: incongruent	PA intervention improved cognitive performance across the congruent and incongruent conditions (i.e., general effects).

PA: physical activity; EF: executive function.

**Table 2:** Hierarchical multiple regression predicting the pre–post change in cognitive performance.

	Step 1		Step 2	
Variables	β	95% CI	β	95% CI
Group	0.09 *	0.04 to 0.15	0.09 *	0.04 to 0.14
Baseline Performance	−0.57 *	−0.064 to −0.50	−0.56 *	−0.63 to −0.48
Baseline Performance^2^	0.06 *	0.002 to 0.13	0.10 *	0.02 to 0.17
EF Condition	−0.17 *	−0.22 to −0.11	−0.17 *	−0.23 to −0.11
Group × Baseline Performance			−0.07 *	−0.14 to −0.003
Group × Baseline Performance^2^			0.03	−0.03 to 0.09
Group × EF Condition			−0.03	−0.08 to 0.03
Baseline Performance × EF Condition			0.07 *	0.008 to 0.14
Baseline Performance^2^ × EF Condition			−0.003	−0.08 to 0.07
Δ*R*^2^	0.33 *		0.01 *	

*Note:* Cognitive performance was converted to z-score after controlling for age and sex in each study; group is coded as control = 0, intervention = 1; EF condition is coded as lower EF condition = 0, higher EF condition = 1; CI = confidence interval; * *p* < 0.05. Step 3 did not reach significance (Δ*R*^2^ = 0.002, *p* = 0.30). Baseline Performance^2^ = square value of Baseline Performance.
